# Exostotic Compression of the Heart Mimicking Angina: A Rare Thoracic Osteochondroma

**DOI:** 10.1016/j.atssr.2026.02.020

**Published:** 2026-03-05

**Authors:** Ashley M. Aldridge, Peter J. Kneuertz, Desmond M. D’Souza, Robert E. Merritt, Ioana Baiu

**Affiliations:** 1 Division of Thoracic Surgery, Department of Surgery, The Ohio State University Wexner Medical Center, Columbus, Ohio

## Abstract

Primary tumors of the bony chest wall are rare. A young ultramarathon runner presented with angina-like chest pain with normal findings on cardiac workup. Cross-sectional imaging revealed a 4.2-cm lesion abutting the pericardium concerning for an osteochondroma. This case illustrates how exostotic compression from a rib lesion can mimic angina and prompt extensive cardiac evaluation while the true cause remains unrecognized, highlighting the need for timely cross-sectional imaging and early surgical intervention. Video-assisted thoracoscopic surgery confirmed a 4.2-cm mass impinging on the pericardium, and resection with reconstruction provided complete symptom resolution and a return to running.

Primary chest wall tumors are uncommon lesions, representing <2% of all new primary neoplasms. Despite their rarity, they often pose a diagnostic challenge because presenting symptoms are nonspecific and may overlap with far more common cardiopulmonary conditions. Most patients initially seek evaluation for chest pain, which is reported across multiple series as the dominant presenting complaint.^1^, ^2^, ^3^ These tumors are broadly categorized by tissue of origin (soft tissue or bone), a distinction that helps guide both diagnostic evaluation and oncologic management. Soft tissue masses are frequently painless and discovered incidentally, whereas bone tumors are commonly manifested with localized, reproducible pain.^4^ Within the spectrum of chest wall neoplasms, primary bone tumors of the chest wall predominantly arise from the ribs, accounting for nearly 85% in some series, of which most, 88%, are malignant.^1^ The likelihood of malignancy is influenced by age, with higher malignant potential at the extremes of age.^4^ Here we describe an unusual presentation of a symptomatic osteochondroma of the rib that produced angina-like chest pain and was initially misinterpreted as a pulmonary nodule and attributed to anxiety. This case highlights the importance of maintaining a broad differential diagnosis in evaluating atypical chest pain, particularly when symptoms are positional or reproducible, and underscores the value of careful imaging review in avoiding misdiagnosis.

A 34-year-old otherwise healthy ultramarathon runner and never-smoker presented with intermittent tachycardia, dyspnea, and progressive left-sided chest pain radiating to his back, neck, and arm, accompanied by left arm paresthesias. Electrocardiogram and serial troponins were unremarkable. A chest radiograph demonstrated a left midlung nodule thought to represent a partially calcified granuloma. His symptoms were attributed to anxiety, and he was prescribed vortioxetine (Trintellix) and trazodone. As his symptoms persisted, he was evaluated by a cardiologist. Findings on transthoracic echocardiography and nuclear myocardial perfusion stress testing were normal, showing an ejection fraction of 55% to 60%, excellent exercise capacity, and no evidence of ischemia. His symptoms progressively worsened to the point that he could no longer engage in his usual activities because of exertional pain and an inability to lie on his left side. Nearly a year after his initial presentation, a chest computed tomography scan was obtained to further characterize the presumed pulmonary nodule. Computed tomography revealed an elongated anterior left third rib osteochondroma extending into the thoracic cavity, measuring 4.2 cm with a cartilaginous cap, and tracking along an incomplete left upper lobe horizontal fissure (Figure 1). The patient was reassured that this represented a benign lesion; however, because of ongoing symptoms, he self-referred to the thoracic surgery clinic.

**Figure 1:**
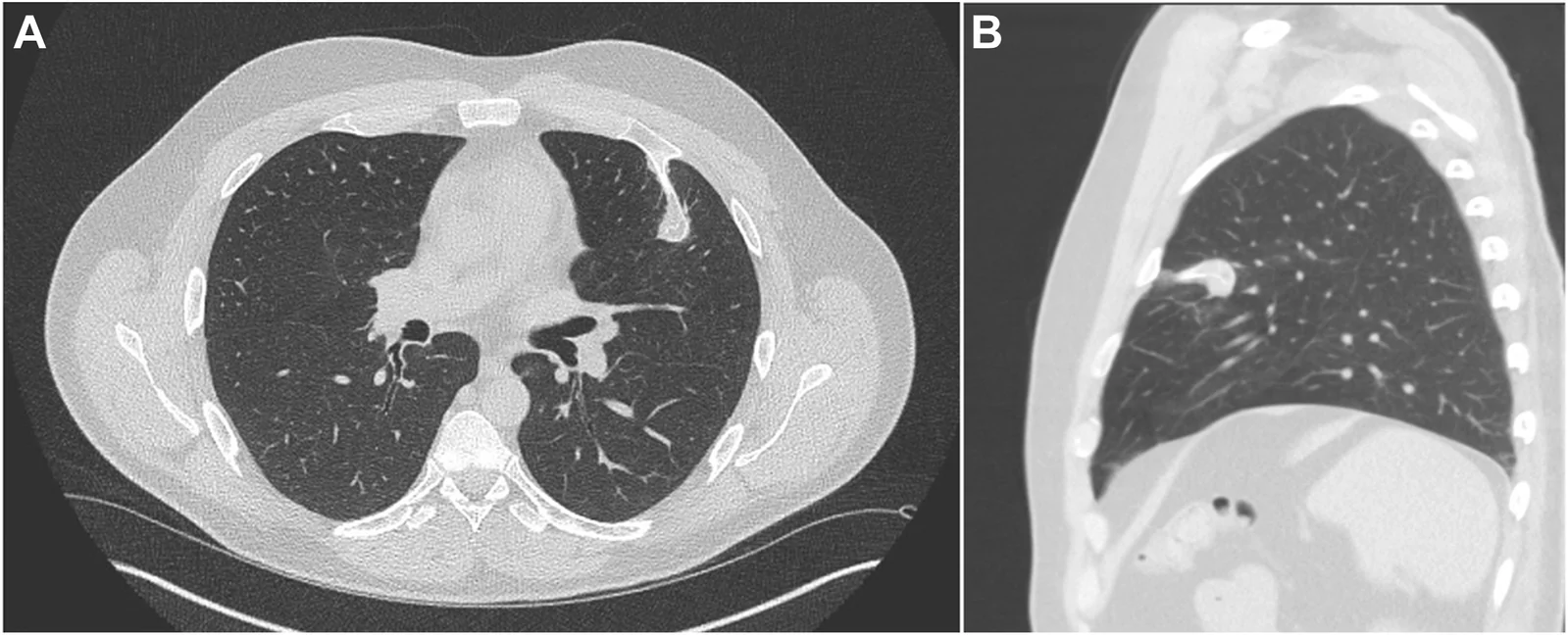
Computed tomography scan of lesion. (A) Axial view of anterior left third rib elongated lesion extending into intrathoracic cavity/lung. (B) Sagittal view of anterior left third rib lesion.

Given the severity of his symptoms and imaging findings, operative intervention was recommended. Diagnostic left video-assisted thoracoscopic surgery through a 5-mm incision in the seventh midaxillary intercostal space was performed to mark the proximal and distal extent of the involved rib using spinal needles and to exclude additional intrathoracic disease (Figure 2A). A 4.2-cm protuberant rib lesion with a cartilaginous cap was identified, impinging on the pericardium and abutting the lung—correlating with the patient's angina-like symptoms (Figures 2A, 2B). A 5-cm left anterior minithoracotomy was then performed over the defined area. The third rib was carefully dissected to preserve the intercostal neurovascular bundle and resected with 2-cm proximal and distal margins using the Sonopet (Stryker) device (Figure 3). Although chest wall reconstruction is typically unnecessary after a single-rib resection, the residual rib ends were noted to angulate downward toward the pericardium. As such, the chest wall was stabilized with a titanium rib plate secured with locking screws. The patient recovered well and was discharged on postoperative day 3. Final pathologic examination confirmed a benign osteochondroma. His chest pain completely resolved, and he was able to return to running without limitation.

**Figure 2:**
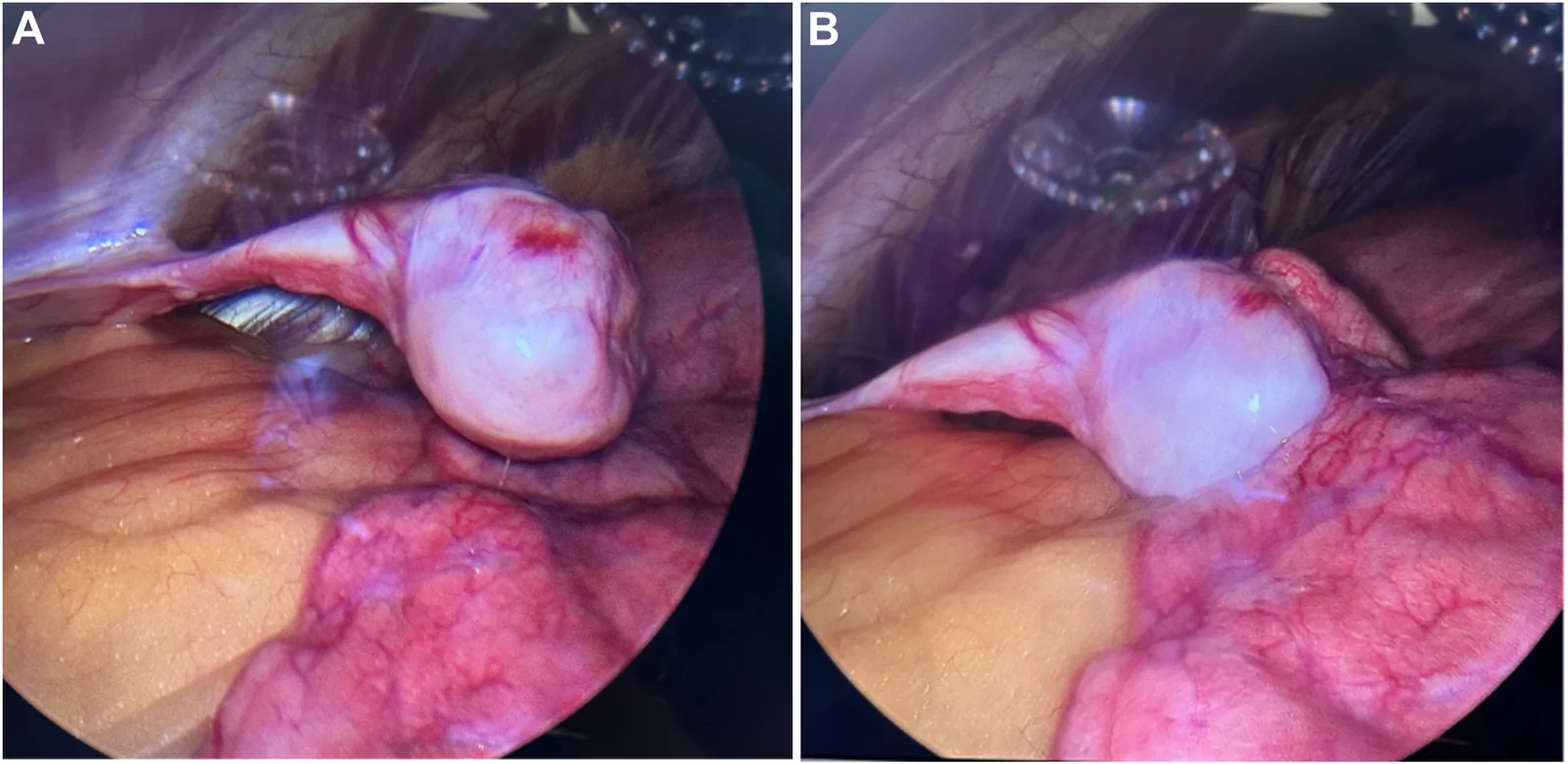
Anterior lesion of the third rib. (A) Diagnostic video-assisted thoracoscopic surgery was performed first to identify the precise location of the mass and relation to other structures. (B) The mass was close to—nearly impinging on—the heart.

**Figure 3:**
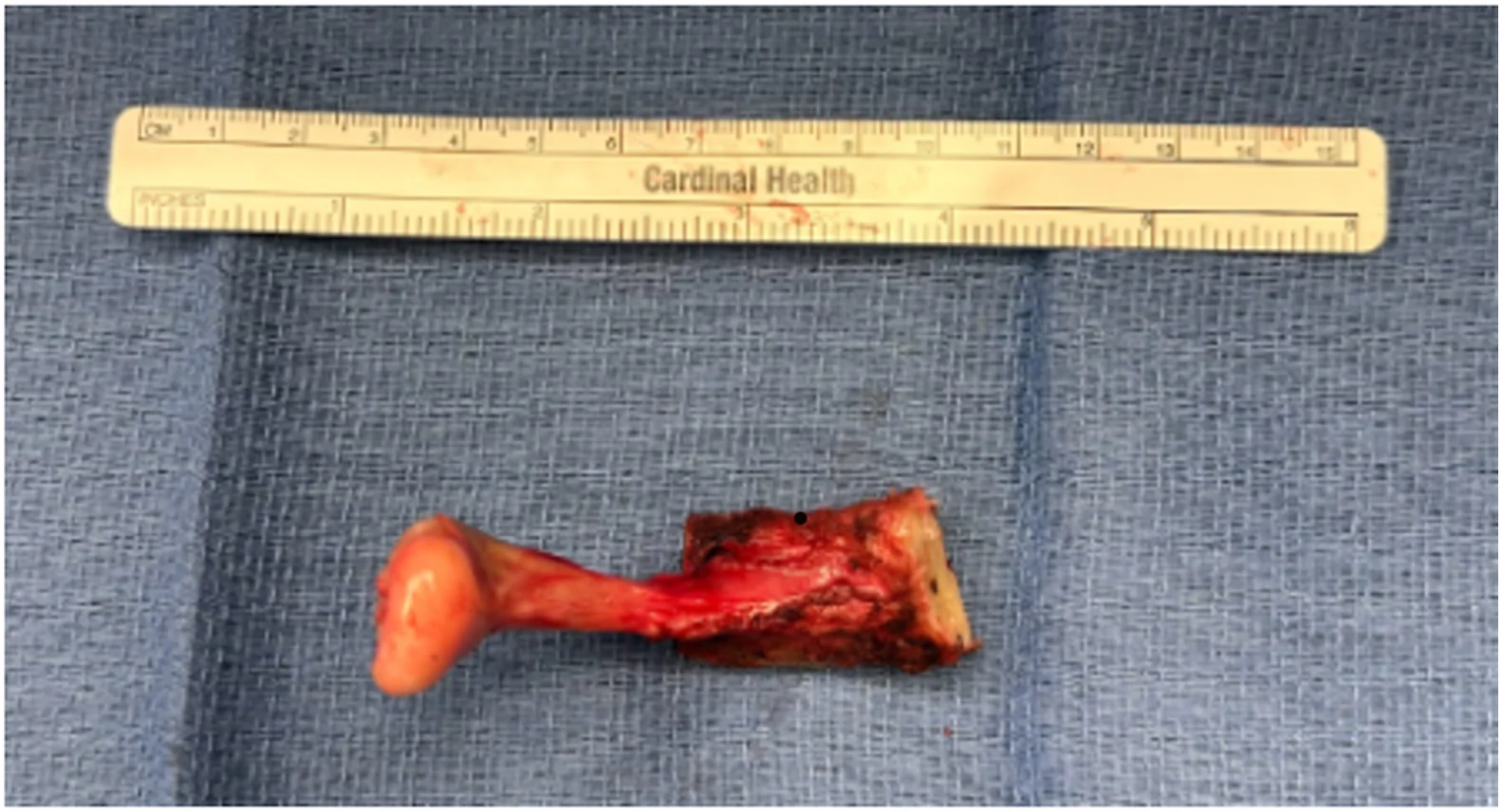
Extracorporeal specimen sent for pathologic examination.

## Comment

Although primary chest wall tumors are uncommon,^1^, ^2^, ^3^, ^4^ their clinical presentation ranges from complete absence of symptoms to significant chest pain. This case illustrates how a patient’s persistent, quality of life–limiting symptoms led to an extensive cardiac evaluation and misdiagnoses of anxiety and a presumed lung nodule, neither of which was correct. Although the eventual diagnosis was established on cross-sectional imaging, the patient was falsely reassured that a benign rib lesion did not warrant intervention. In reality, exostotic compression of the heart from a rib osteochondroma was the source of his symptoms, and surgical resection resulted in complete resolution.

Osteochondromas account for 30% to 50% of benign bone tumors of the chest wall.^4^ They arise from the metaphyseal region of the rib, most commonly at the costochondral junction.^1^^,^^4^ These lesions are typically manifested in the second decade of life, and men are affected 3 times more often than women.^1^ On radiographic examination, osteochondromas appear as lobulated, pedunculated, or sessile projections of cortical bone with a cartilaginous cap. They are often poorly visualized on chest radiography, although calcification within the cartilaginous cap may occasionally permit detection, as in our case.^1^^,^^2^ Osteochondromas are usually round, measuring <9 cm.^4^ A cartilaginous cap thicker than 2 cm raises concern for malignant degeneration.^1^^,^^4^ Because of the risks of pathologic fracture and malignant transformation (1%-2%), these tumors should be resected en bloc as a segment of the rib with grossly negative margins.^1^^,^^2^ The resection should include all or most of the rib involved, a section of any adjacent ribs, and en bloc resection of any attached structures, regardless of tumor shape or attachment pattern.^4^ For this reason, all osteochondromas should be regarded as potentially malignant and managed surgically with wide local resection.^1^^,^^3^ Recurrence can develop if the cartilaginous cap is not completely resected.^1^ Early diagnosis and aggressive surgical resection of most primary chest wall tumors are associated with excellent long-term outcomes.^1^ In this case, the markedly anterior location of the tumor with impingement on the pericardium rendered a purely minimally invasive resection technically challenging with current surgical instrumentation. Nonetheless, an initial diagnostic video-assisted thoracoscopic surgery approach is still valuable to address any intrathoracic adhesions and to delineate proximal and distal margins of resection, thereby minimizing the length of the subsequent open incision. In addition, the tumor’s location resulted in significant angulation of the proximal and distal rib ends after resection, necessitating chest wall reconstruction with a titanium plate. This consideration was particularly important in a young, healthy, active patient, in whom preservation of postoperative chest wall stability and minimization of dynamic mechanical defects are paramount.

In conclusion, bone tumors of the chest wall, although uncommon, may produce symptoms that closely mimic cardiopulmonary disease, leading to diagnostic delays and misattribution of symptoms. As illustrated in this case, exostotic compression from a rib lesion can be manifested as angina-like chest pain, prompting extensive cardiac evaluation while the underlying cause remains unrecognized. Recognition of this potential presentation is critical as delayed diagnosis may prolong patient morbidity and obscure the underlying pathologic process. This case underscores the importance of maintaining a broad differential in evaluating atypical chest pain, particularly when it is positional or reproducible, pursuing definitive cross-sectional imaging, and considering early surgical referral when symptoms are unexplained or refractory to avoid misdiagnosis. Timely and complete resection not only establishes the diagnosis and excludes malignancy but also provides durable symptom relief and restoration of quality of life.
